# A large-scale MEG and EEG dataset for object recognition in naturalistic scenes

**DOI:** 10.1038/s41597-025-05174-7

**Published:** 2025-05-23

**Authors:** Guohao Zhang, Ming Zhou, Shuyi Zhen, Shaohua Tang, Zheng Li, Zonglei Zhen

**Affiliations:** 1 Beijing Key Laboratory of Applied Experimental Psychology, Faculty of Psychology, Beijing Normal University, Beijing, 100875 China; 2 State Key Laboratory of Cognitive Neuroscience and Learning & IDG/McGovern Institute for Brain Research, Beijing Normal University, Beijing, 100875 China; 3 Department of Systems Science, Faculty of Arts and Sciences, Beijing Normal University, Zhuhai, 519087 China; 4 Department of Psychology, Faculty of Arts and Sciences, Beijing Normal University, Zhuhai, 519087 China

**Keywords:** Perception, Object vision

## Abstract

Neuroimaging with large-scale naturalistic stimuli is increasingly employed to elucidate neural mechanisms of object recognition in natural scenes. However, most existing large-scale neuroimaging datasets with naturalistic stimuli primarily rely on functional magnetic resonance imaging (fMRI), which provides high spatial resolution but is limited in capturing the temporal dynamics. To address this limitation, we extended our Natural Object Dataset-fMRI (NOD-fMRI) by collecting both magnetoencephalography (MEG) and electroencephalography (EEG) data from the same participants while viewing the same naturalistic stimuli. As a result, NOD contains fMRI, MEG, and EEG responses to 57,000 naturalistic images from 30 participants. This enables the examination of brain activity elicited by naturalistic stimuli with both high spatial resolution (via fMRI) and high temporal resolution (via MEG and EEG). Furthermore, the multimodal nature of NOD allows researchers to combine datasets from different modalities to achieve a more comprehensive view of object processing. We believe that the NOD dataset will serve as a valuable resource for advancing our understanding of the cognitive and neural mechanisms underlying object recognition.

## Background & Summary

An important goal of visual neuroscience is to understand the neural mechanisms by which the human brain rapidly and effortlessly recognizes various objects in natural scenes encountered in daily life. To this end, the naturalistic paradigm is becoming increasingly popular in visual neuroscience, which utilizes complex, dynamic, and diverse naturalistic stimuli^1–6^. Emerging evidence shows naturalistic stimuli, which encapsulate a wide variety of visual features and cognitive contexts, can induce highly reproducible brain responses and thus help researchers accurately characterize the spatiotemporal activation patterns of the brain across a wide range of spatiotemporal scales^7–10^. In recent years, a series of high-quality, large-scale brain imaging datasets based on naturalistic visual stimuli have been released to propel the field of human visual neuroscience, including datasets that use discrete natural images or clips as stimuli, such as Natural Scenes Dataset (NSD)^11^, Natural Object Dataset (NOD)^12^, Human Action Dataset (HAD)^13^, Bold Moments Dataset (BMD)^14^, and THINGS^15^, as well as datasets that use continuous movies as stimuli, such as The Grand Budapest Hotel^16^, Forrest Gump^17^. These datasets provide valuable resources for exploring the brain organization and coding principles of the visual cortex in processing naturalistic stimuli. Most of these datasets are functional magnetic resonance imaging (fMRI) data, and only a few datasets collect magnetoencephalography (MEG) or electroencephalography (EEG) data using naturalistic visual stimuli.

Functional MRI excels at capturing spatial activity patterns because of its high spatial resolution. Yet, the low temporal resolution limits its ability to accurately characterize the neural spatiotemporal dynamics of object recognition in naturalistic scenes. In contrast to fMRI, MEG and EEG record neural magnetic and electrical activity with millisecond-level temporal resolution, making them ideal for examining the rapid temporal dynamics of neural activity^18,19^. Moreover, MEG and EEG show complementary sensitivity profiles and field patterns^20–22^. MEG is more sensitive to tangential sources located in the sulci, while EEG is more sensitive to radial sources located in the gyri. However, to our knowledge, only two publicly available datasets collect both fMRI and M/EEG data under identical large-scale naturalistic stimuli: the StudyForest datasets, which include fMRI^23^ and MEG^17^ data with the Forrest Gump movie as stimuli. THINGS^15^, as the first dataset to include fMRI, MEG, and EEG modalities in response to natural images, has served as an excellent reference template for investigating natural object representations in human brain with different techniques. However, given the complex and rich information contained in natural images, more data are still needed to accurately characterize the spatiotemporal patterns of brain activity in response to the natural stimuli.

To address this gap, we extended the previously collected NOD-fMRI dataset by acquiring MEG and EEG data from the same participants and stimuli, making NOD a multimodal dataset integrating both high temporal resolution (M/EEG) and high spatial resolution (fMRI). Inheriting from NOD-fMRI, the NOD’s MEG and EEG aims to accurately capture brain activation patterns and to characterize both within-individual differences elicited by various naturalistic stimuli and inter-individual variability from similar stimuli. Firstly, to accurately capture the differences in neural activation patterns in response to diverse stimuli within individuals, we initially collected MEG and EEG data from 9 participants viewing 4,000 images from ImageNet. Secondly, to better capture inter-individual differences, we collected EEG and MEG data from an additional 21 participants who viewed at least 1,000 images from ImageNet. This expansion resulted in a comprehensive dataset (N = 30) that is particularly effective at characterizing variability across individuals than other publicly available naturalistic neuroimaging datasets, which typically include fewer than 10 participants.

Compared to the landmark multimodal naturalistic neuroimaging dataset THINGS, the NOD exhibits several distinct features. Firstly, the NOD dataset includes EEG, MEG, and fMRI data collected from the same participants, whereas the EEG and fMRI/MEG data in the THINGS dataset are derived from separate participant groups. Secondly, the NOD dataset comprises a larger number of participants than the THINGS dataset. We anticipate that including MEG and EEG data for the NOD dataset will facilitate precise characterization of the human brain’s neural activity patterns involved in processing objects within naturalistic scenes and advance our understanding of the neural mechanisms underlying human object recognition.

## Methods

### Participants

The participants in NOD-MEG and NOD-EEG experiments were recruited from the same cohort that had previously participated in the NOD-fMRI experiment, thereby ensuring that the fMRI, EEG, and MEG data were obtained from the same individuals. A total of 30 healthy participants (18 females, mean age ± standard deviation [SD], 21.23 ± 1.98 years) took part in the NOD-MEG experiment. Among them, 19 participants (8 females, mean age ± SD, 21.26 ± 2.00 years) were involved in the NOD-EEG experiment. All participants had normal or corrected-to-normal vision, reported no history of psychiatric or neurological disorders, and provided written informed consent for both their participation and the sharing of their anonymized data. The Institutional Review Board of Beijing Normal University approved the study (approval number: ICBIR_A_0111_001_02).

### Experiment design

#### Stimuli

Both the NOD-MEG and NOD-EEG experiments used the same stimuli as the NOD-fMRI data. Specifically, a three-stage procedure was utilized to select 60,000 candidate stimuli from ImageNet Large Scale Visual Recognition Challenge 2012 (ILSVRC2012)^24^, which contains more than one million images annotated of the 1000 object categories. Initially, 60 images were randomly sampled for each category, with criteria requiring a square aspect ratio (≈1) and high resolution (>100,000 pixels). These images were then visually inspected to identify blurred or mislabelled instances. Finally, the detected improper images were replaced with those meeting the initial criteria, ensuring the integrity and quality of the dataset. Then, the 60,000 images were used as the stimuli superset from which images were selected for the MEG and EEG experiments.

#### MEG experiment

**Fig. 1:**
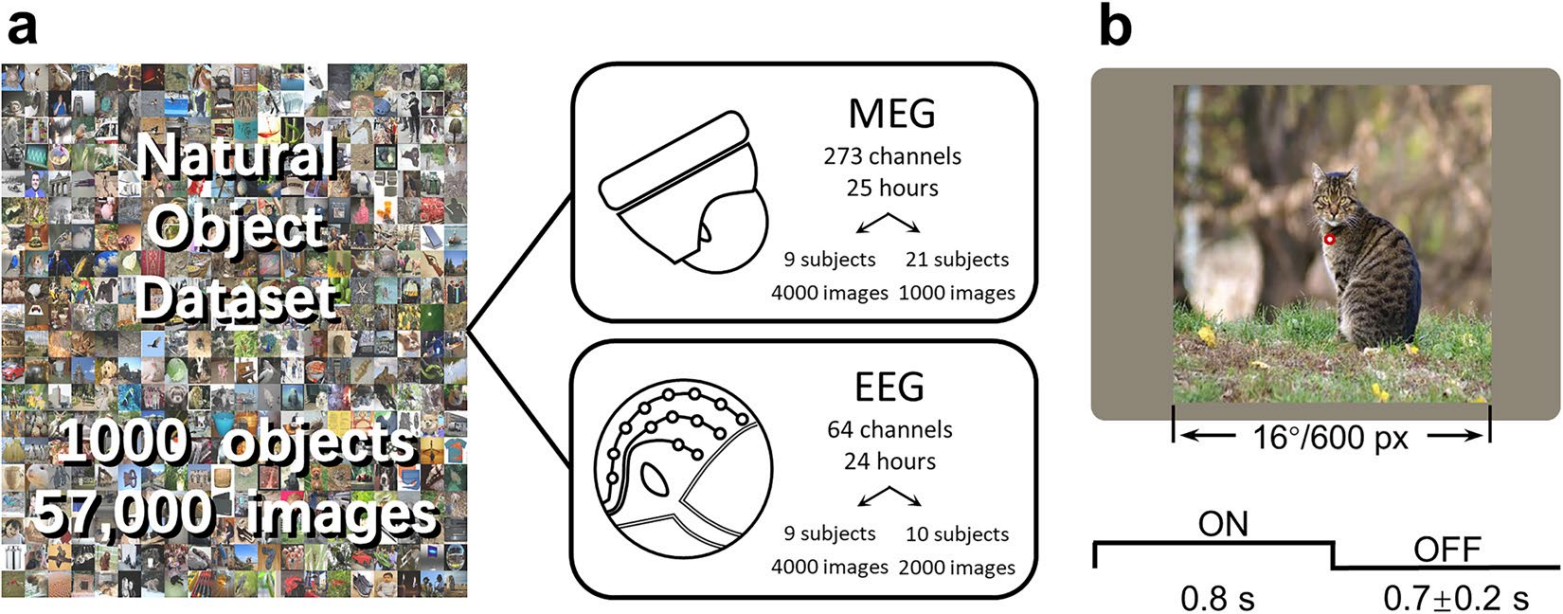
Design of the Natural Object Dataset (NOD) experiments. (**a**) The stimuli used in the NOD-M/EEG experiment are derived from the ImageNet database, which comprises 1,000 object categories, with 57 images per category. In total, 25 hours MEG data (273 channels) were acquired from 30 participants and 24 hours EEG data (64 channels) were acquired from 19 participants. (**b**) In the experiments, an image was presented for 0.8 seconds, followed by a fixation cross for 0.7 seconds, with a jitter of ± 0.2 seconds.

Each trial of NOD-MEG lasted approximately 1500 ms, with the stimulus presented for 800 ms, followed by a variable fixation period of 700 ± 200 ms. The stimuli were loaded using Psychophysics Toolbox Version 3 (PTB-3) in MATLAB 2016 (MathWorks, Natick, Massachusetts, USA), generated by a standard PCI graphics card (GeForce GT620; NVIDIA, Santa Clara, California, USA), and then projected onto a screen with a resolution of 1024 × 768 pixels via a DLP projector (NP63+; NEC, Tokyo, Japan). Participants viewed the visual stimuli on the projection screen via a mirror reflection. Each stimulus was 600 × 600 pixels in size (visual angles = 16°; viewing distance = 700 mm, Fig. 1b). Each run of the experiment consisted of 200 trials, lasting approximately 390 seconds. In the experiment, participants were asked to press a button to indicate whether the object presented in the image was animate. Among the 30 participants, 9 completed 4 MEG sessions, each viewing a total of 4000 unique images (four images/category). These four sessions comprised ses-ImageNet01 (2 runs), ses-ImageNet02 (2 runs), ses-ImageNet03 (8 runs), and ses-ImageNet04 (8 runs). The remaining 21 participants completed only 1 MEG session, namely ses-ImageNet01 (5 runs), and each viewed 1000 unique images (one image/category). Therefore, we collected a total of 25 hours of MEG data on 30 participants with 57000 images as stimuli (Fig. 1a). Additionally, we also collected MRI structural images for each participant to facilitate source localization of MEG signals.

#### EEG experiment

Each trial of NOD-EEG lasted approximately 1500 ms, with the stimulus presented for 800 ms, followed by a variable fixation period of 700 ± 200 ms. The stimuli were loaded using Psychophysics Toolbox Version 3 (PTB-3) in MATLAB 2016 (MathWorks, Natick, Massachusetts, USA), generated by a standard PCI graphics card (GeForce GT620; NVIDIA, Santa Clara, California, USA), and then displayed on a screen with a resolution of 1440 × 960 pixels. Each stimulus was 600 × 600 pixels in size (visual angles = 16°; viewing distance = 700 mm, Fig. 1b). Each run of the experiment consisted of 125 trials, lasting approximately 190 seconds. Consistent with the MEG experiment, participants were required to press a button to indicate whether the presented object was animate. Among the 19 participants, 9 completed 4 EEG sessions (ses-ImageNet01-04), each consisting of eight runs and 4,000 unique images (four images/category) viewed per participant. The remaining 10 participants completed 2 EEG sessions (ses-ImageNet01-02), also with eight runs per session and 2,000 unique images (two images/category) viewed per participant. As a result, we collected a total of 24 hours of EEG data from 19 participants with 56000 images as stimuli (Fig. 1a).

### Data acquisition

#### MEG recordings

MEG data were recorded using a 275-channel whole-head axial gradiometer DSQ3500 MEG system (CTF, Canada) at the Institute of Biophysics, Chinese Academy of Sciences, Beijing, China. Three channels (MLF55, MRT23, and MRT16) were not used due to equipment malfunctions. The MEG signal sampling rate was 1200 Hz, without the use of online filters during acquisition. To accurately synchronize stimuli with neural signals, we used real-time markers to record the sampling points of stimulus presentations and participant responses, with all marker information stored in the UPPT001 stimulus channel of the MEG data. At the start of each session, three head position indicator coils were attached to the participant’s nasion (NAS), left preauricular point (LPA), and right preauricular point (RPA) to measure their head position in the MEG helmet. A custom chin rest was used during data collection to minimize potential head movement of the participants.

#### EEG recordings

EEG data were recorded using a 64-channel EEG system with an international 10–20 system (NeuroScan, USA) at the Faculty of Psychology, Beijing Normal University, Beijing, China. The EEG signal was recorded using Curry Neuroimage 8 with sampling rate as 500 Hz, amplified by a SynAmps 2 amplifier (Compumedics NeuroScan). The entire EEG acquisition process was conducted in an electromagnetically shielded room, and participants were instructed to minimize movement during data acquisition.

#### Structural MRI

Structural T1w images were acquired for anatomical reference with a three-dimensional magnetization-prepared rapid acquisition gradient echo sequence (1 slab; 208 sagittal slices; FOV, 256 × 256 mm; slice thickness, 1 mm; isotropic voxel size, 1 × 1 × 1 mm; TR, 2530 ms; TE, 2.27 ms; TI, 1100 ms; and flip angle, 7°) using a Siemens MAGNETOM Prisma 3 T MRI scanner with a 64-channel phased-array head coil at the Imaging Center for Brain Research, Beijing Normal University, Beijing, China .

**Fig. 2:**
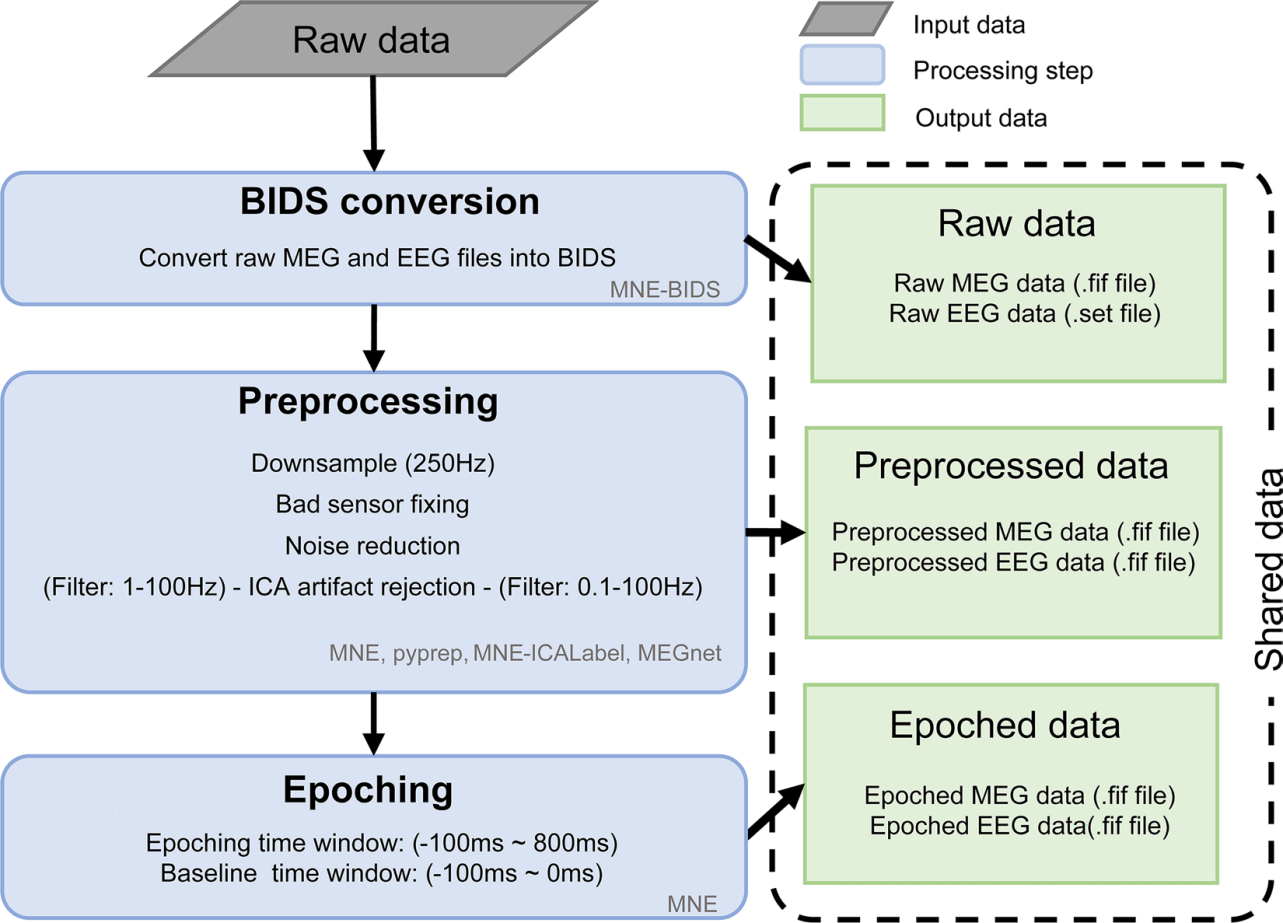
Overview of data processing pipeline and the shared data. The raw M/EEG data were primarily processed using the MNE software suite. The shared data includes the raw, preprocessed, and epoched data.

#### Data preprocessing

Both MEG and EEG data were preprocessed offline by combining different tools including MNE-BIDS^25,26^, MNE-Python^27,28^, MEGnet^29^, MNE-ICALabel^30^, meegkit^31^ and pyprep^32^. The data processing workflow generally included the following six steps, after which the raw data, preprocessed time series (preprocessed data), and trial-segmented (epoched) data for each participant were shared (Fig. 2).

1. BIDS conversion. The raw MEG and EEG data were first converted to Brain-Imaging-Data-Structure (BIDS) format. During this process, we manually checked the data integrity and wrote participant information, experiment time, and other details into the BIDS structure.
2. Downsample. M/EEG data were downsampled to 250 Hz to reduce the computational burden of downstream analysis.
3. Bad sensor fixing. In M/EEG, sensors can sometimes be too noisy or quiet, making it difficult to record brain signals. We used established automatic bad channel detection algorithms (Maxwell^27^ for MEG, RANSAC^32^ for EEG) to detect bad channels in each run of M/EEG. No bad channels were found in the MEG data, while 1.34 bad channels (SD = 2.32) on average were detected per EEG data run. The bad channels were then corrected using interpolation algorithms (spherical splines interpolation^33^ for EEG, field map interpolation^27^ for MEG).
4. Noise reduction. Due to the high sensitivity of M/EEG recordings, weak brain signals are often mixed with environmental noise. The Zapline algorithm^34^ was used to reduce power line noise (50 Hz) in both MEG and EEG (Fig. 3a). Besides, the CTF’s third-order gradient compensation algorithm was applied to reduce noise in the MEG data, and the average of all EEG channels was used to re-referencing the EEG data.

**Fig. 3:**
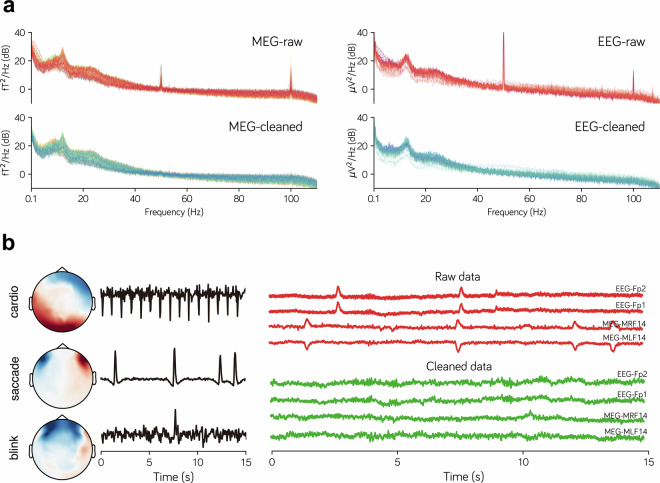
The effects of line noise reduction and ICA artifacts removal from a sample participant (i.e., sub01). (**a**) The Power Spectral Density (PSD) of the raw MEG and EEG data shows line noise at 50 Hz and its harmonics (upper panel). The line noise was effectively removed using zapline denoising (lower panel). (**b**) The left panel shows the spatial maps and time series of typical artifact components extracted from M/EEG data via ICA decomposition. The right panel presents the data from two frontal lobe sensors in the M/EEG recordings: the upper section shows the raw signals before artifact rejection, while the lower section presents the cleaned signals following artifact rejection.
5. ICA artifact rejection. Each run of MEG and EEG data was first filtered within the 1–100 Hz range to reduce the impact of low-frequency drifts and high-frequency noise on the ICA decomposition. The data was then decomposed into different independent components using ICA algorithm, and the artifacts components were identified by combining the automatic artifact selection algorithms (MEGnet^29^ for MEG, MNE-ICALabel^30^ for EEG) and manual inspection. Finally, these artifact components were removed on the 0.1–100 Hz time series through regression (Fig. 3b).
6. Epoching. We divided the time window of each trial from −100ms to 800 ms relative to stimulus onset to fully capture the neural response induced by the stimulus. Baseline correction was performed using the time window from 100 ms before onset of the images. Then, all trials for each participant were concatenated to create the participant’s epoch data.

## Data Records

**Fig. 4:**
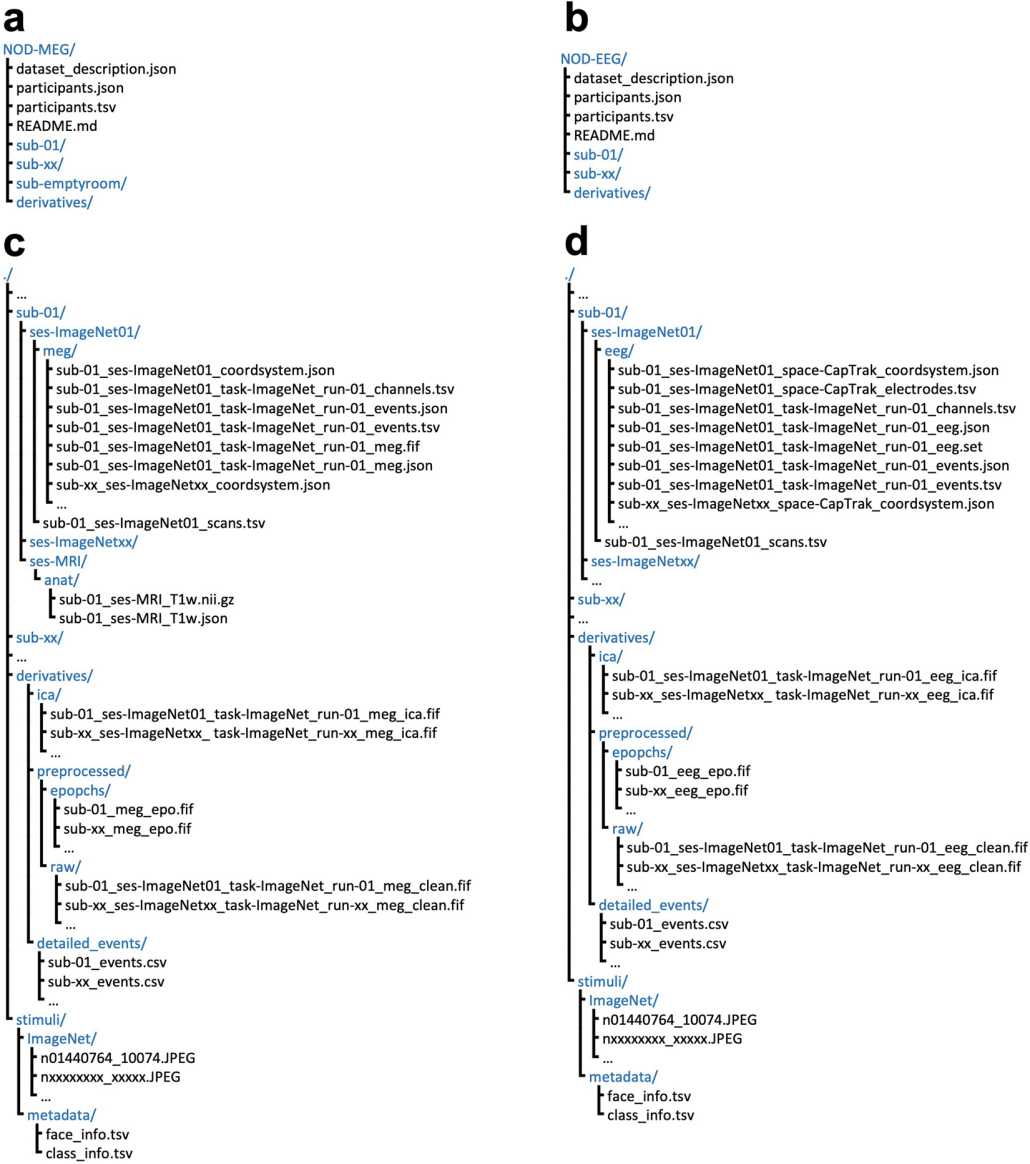
The directory and file structure of the Natural Object Dataset’s M/EEG data. (**a,b**) Overview of the directory and file structure for the MEG and EEG data, respectively. (**c,d**) A detailed view of the file structure from a single sample subject (i.e., sub01) for the MEG and EEG data, respectively.

Both MEG and EEG data were organized into the BIDS format via mne-bids and uploaded to the OpenNeuro public repository^35^. The accession numbers are ds005810^36^ and ds005811^37^ for MEG and EEG data, respectively. In brief, the raw MEG and EEG data from each participant are stored in the “sub-<subID>” directory, while preprocessed data and epoch data are stored in the “derivatives/preprocessed/raw” and “derivatives/preprocessed/epochs” directories, respectively (Fig. 4a,b).

### Stimulus images

The stimuli images used for MEG and EEG are identical and are stored in the “stimuli/ImageNet” directory. Images within the folder are named in the format “<synsetID>_<imageID>.JPEG”, where synsetID is the ImageNet category information, and imageID is the unique number for the image within that category. The image metadata including category information is available in the table files under “stimuli/metadata”.

### Raw data

Raw MEG and EEG data are stored in BIDS format in the NOD-MEG and NOD-EEG directories, respectively (Fig. 4c,d). Each participant’s directory contains multiple session folders, designated as “ses-<sesID>”, which in turn include “meg” or “eeg” data folders. The “ses-MRI/anat” directory contains the participant’s structural MRI images. The comprehensive trial information for each participant is documented in the file “ derivatives/detailed_events/sub-<subID>_events.csv” in which each row corresponds to a trial, and each column contains metadata for that trial, including the session and run ID, category information of the stimuli, and participant response.

### Preprocessed data

The full-time series data of preprocessed MEG and EEG are archived in the “derivatives/raw” directory, named “sub-<subID>_ses-<sesID>_task-ImageNet_run-<runID>_<meg/eeg>_clean.fif”. The epoch data derived from preprocessed MEG and EEG are stored within the “derivatives/epochs” directory. In this directory, all data for each participant are concatenated into a single file, named as “sub-<subID>_epo.fif”. The trial information within each participant’s epochs data can be accessed via the metadata of the epochs data, which are aligned with the content of the participant’s “sub-<subID>_events.csv” file.

## Technical Validation

### Recognition accuracy

**Fig. 5:**
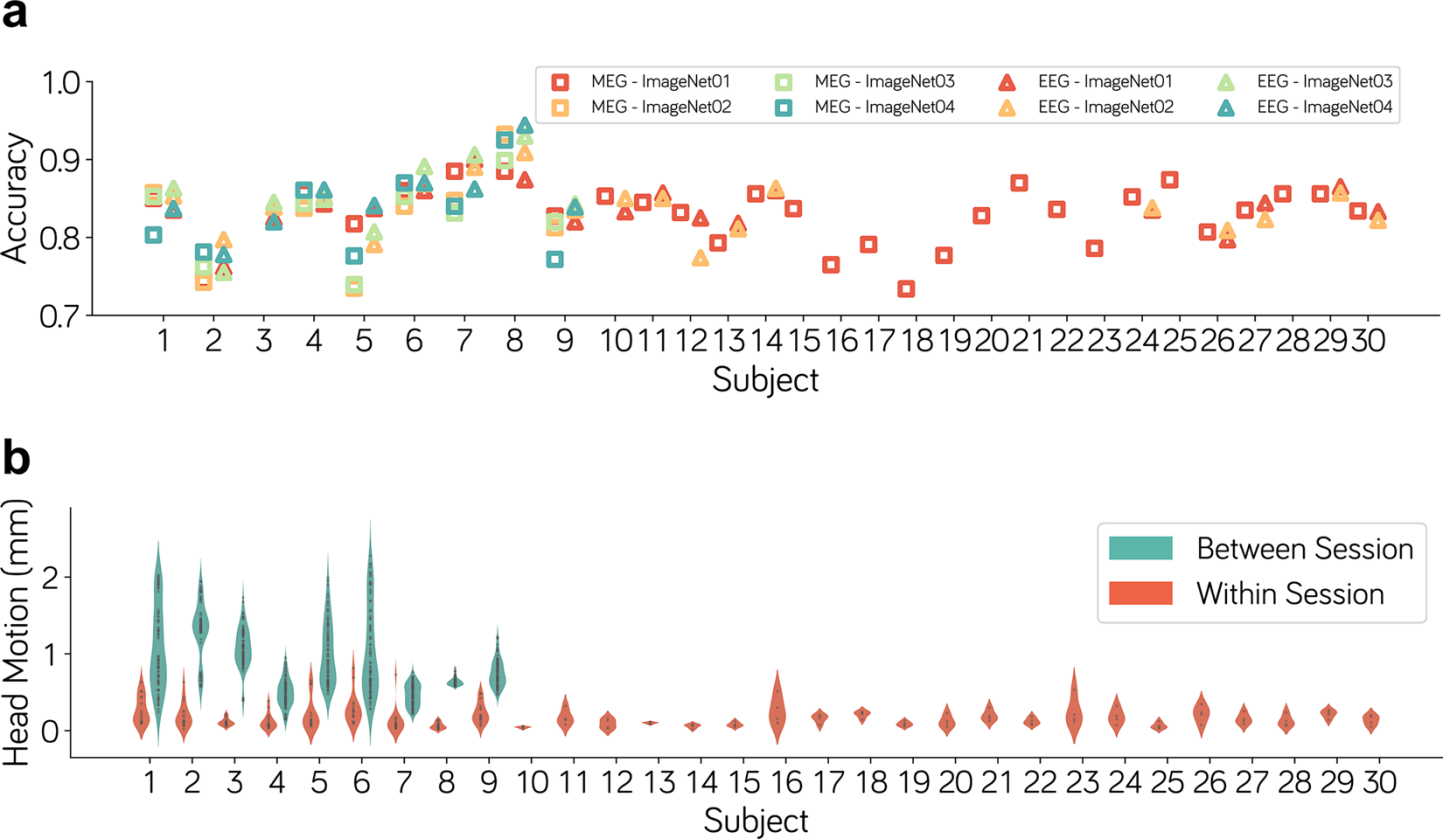
Basic quality metrics for M/EEG datasets. (**a**) The mean recognition accuracy for each subject in each session of the MEG and EEG experiments. (**b**) Quantification of MEG head motion. The within-session motion is measured as the Euclidean distance between head coil positions across runs, while the between-session motion is evaluated as the Euclidean distance between head coil positions across different sessions.

Participants’ understanding and active engagement in the experimental task are crucial for the success of the neuroscience experiment. For this, the recognition accuracy for each participant in each session was calculated to evaluate their task engagement. The average recognition accuracy for all participants was 83.69%, with no recognition accuracy lower than 70% in all sessions (Fig. 5a). This suggests that participants were effectively engaged in the task of determining the animacy of the presented images.

### Head motion

Changes of head position in MEG recordings can significantly impact the data quality of MEG. As during MEG data collection, the coordinates of three reference points on the human brain (i.e., NAS, LPA, and RPA) are recorded, we can quantify intra-session head motion (between runs) for each participant by calculating the distance between initial run positions, and evaluate inter-session head motion by calculating the average positional change across sessions. As shown in Fig. 5b, all participants exhibited consistently low head motion (median = 0.14 mm) within sessions. Although head motion between sessions was slightly higher, it remained at a very low level overall (median = 1.01 mm), indicating participants show good control of head motion within the dataset. Since EEG data collection does not record the head position, the head motion in EEG recordings was not assessed.

### Temporal dynamics of face representation

**Fig. 6:**
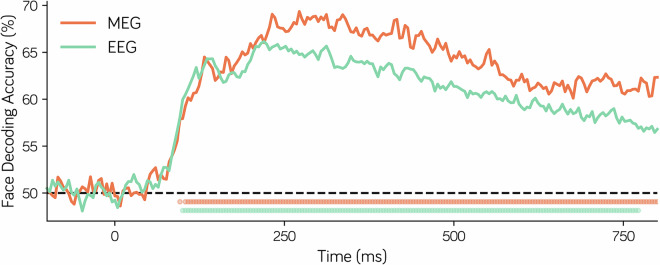
Decoding analysis of face vs. object based on the spatial pattern at each time point measured by MEG and EEG shows temporal dynamics of face representation. The line represents the mean decoding accuracy across all subjects, and the dots below indicate time points where the decoding accuracy is significantly larger than chance level (>50%).

A large body of research has demonstrated that MEG or EEG signals originating from the occipitotemporal lobe can effectively characterize the temporal dynamics of object category representation^38–47^. Here, we attempt to replicate findings from previous studies on the temporal dynamics of face representation using M/EEG signals from the occipitotemporal lobe (MEG 108 sensors, EEG 16 sensors). Specifically, we constructed a linear support vector machine (SVM) with the spatiotemporal patterns from occipitotemporal sensors at each time point as the input to perform face versus object decoding. To this end, we first used InsightFace^48^ to label the stimuli image set into face or object categories. Moreover, to address the imbalance in sample sizes between face and object, we randomly selected samples from the object category to match sample size between object and face. A 10-fold cross-validation procedure was used to estimate classification accuracy. As shown in Fig. 6, both MEG and EEG data reliably decoded the presence of face stimuli in the images and exhibited similar temporal dynamics. However, MEG demonstrated better performance than EEG in decoding face versus object after the onset of decoding timing (~250 ms). This indicates that both MEG and EEG data reliably contain information about object category representation but show different sensitivities.

### M/EEG-fMRI fusion analysis

Leveraging the fusion analysis of the MEG, EEG, and fMRI collected with the same stimuli on the same group of participants, the NOD data provides avenues to explore the spatiotemporal dynamics of object representation. To prove this possibility, we perform a similarity-based fusion analysis of fMRI and M/EEG^49,50^ to examine the temporal dynamics of the ventral temporal cortex (VTC)^51–53^. For the fMRI data, the region of interest (ROI) for the VTC was defined according to the Human Connectome Project Multi-Modal Parcellation (HCP-MMP)^54^, including PIT, V8, VVC, FFC, PH, TE2p, PHA1-PHA3, VMV1-VMV3. The fMRI representational dissimilarity matrix (fMRI-RDM) for the 1000 ImageNet categories was constructed by calculating the correlation distance between the group-level VTC activation patterns (beta maps) obtained by averaging the activation patterns from each category across runs and participants. For the M/EEG data, the occipitotemporal sensors approximately corresponding to the VTC were selected as the ROI (MEG 108 sensors, EEG 16 sensors). The M/EEG-RDMs for the 1000 categories were constructed at each time point by calculating the correlation distance between the group-level category specific spatial patterns which were generated by averaging the activation patterns for each category across all runs and all participants.

**Fig. 7:**
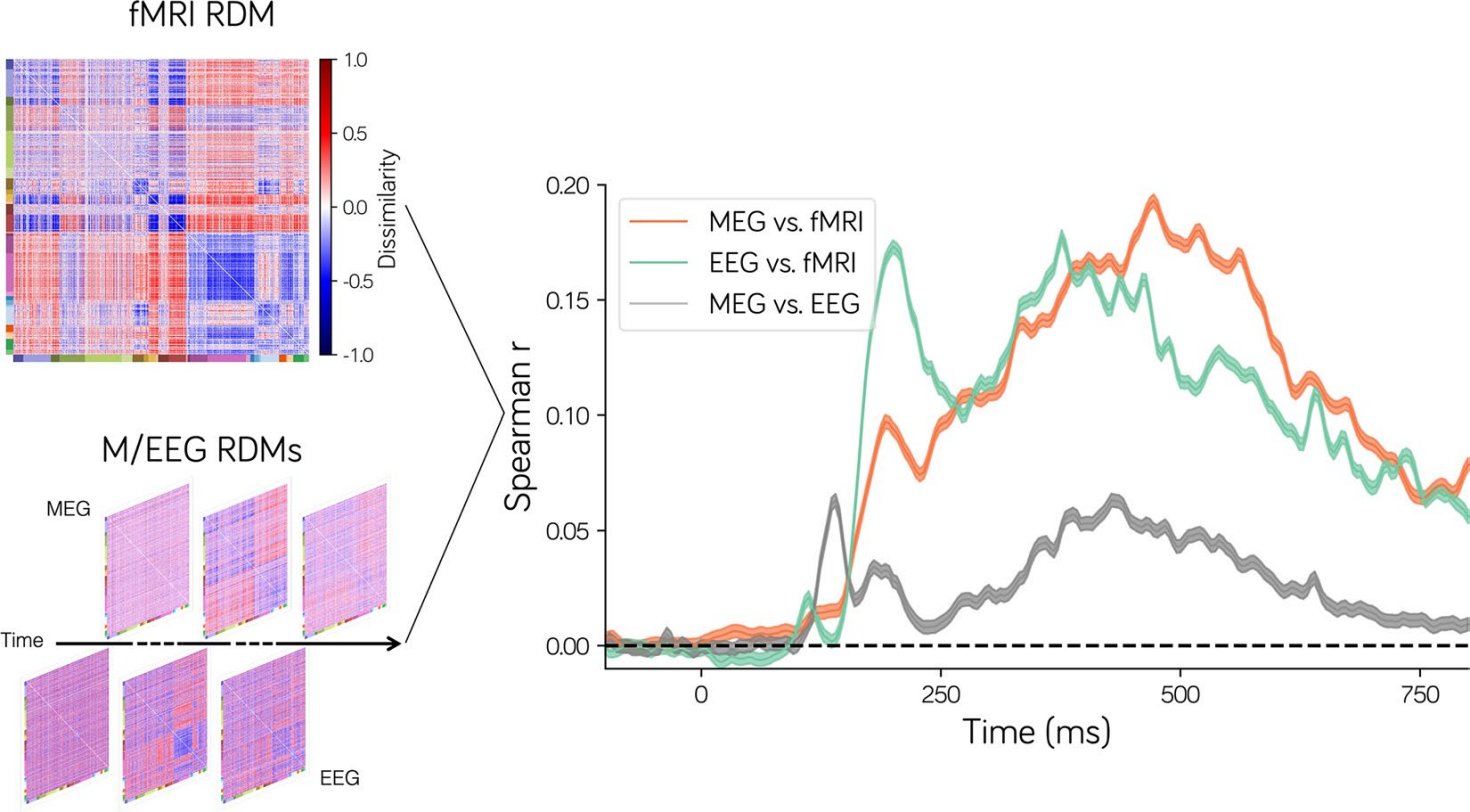
The M/EEG-fMRI fusion analysis reveals the temporal dynamics of the ventral temporal cortex (VTC) in processing naturalistic visual stimuli. The representational dissimilarity matrix (RDM) for the 1000 ImageNet categories was computed with the neural spatial patterns measured by the fMRI, MEG, and EEG, respectively (the left and lower color bars of the RDMs indicate the corresponding ImageNet’s superclass). The Spearman correlation coefficients between M/EEG RDMs and the fMRI RDM were then computed at each time point to integrate the spatiotemporal information from them. The shaded region around the line indicates the 95% confidence interval.

The Spearman correlation coefficients between the RDMs from fMRI, MEG, and EEG were computed at each time point to reveal the temporal dynamics of VTC processing naturalistic stimuli and a non-parametric bootstrap method was used to estimate the significance of the Spearman correlation coefficient. Several findings were revealed (Fig. 7). First, the category representations measured by both MEG and EEG exhibited strong similarity to the category representations of fMRI, indicating that the two modalities shared some common representation information with the fMRI. Second, EEG demonstrated greater representational similarity to fMRI in the early stage (<300 ms), while MEG showed a larger representational similarity to fMRI in the late stage (>400 ms). Finally, the similarity in category representation between MEG and EEG is relatively lower than the similarity between them and fMRI, indicating substantial differences in the origins of MEG and EEG signals or low signal to noise ratio of them. These results suggest that the fusion of fMRI and M/EEG could help us depict the representational dynamics of VTC in object recognition.

## Usage Notes

The present dataset expands upon the NOD-fMRI data by capturing neural activity with MEG and EEG from the same cohort of participants as they viewed the same set of naturalistic image stimuli. The complementary spatial and temporal information from the fMRI, MEG, and EEG data allows for a comprehensive elucidation of brain activity patterns in the processing of naturalistic image stimuli. The multimodal dataset also provides a platform for validating and improving fusion algorithms, helping researchers develop new analytical tools and models in cognitive neuroscience.

Several key points should be noted when utilizing the dataset. Firstly, both participants and stimuli are aligned via unique identifiers (IDs) across the three modalities. That is, the same participant and stimuli ID represent the same participant and stimuli in all of three datasets. Secondly, to help user access the information of each trial, the stimuli information has been embedded directly into the metadata of the mne.Epochs object. Besides, both the preprocessed and epoch data of the M/EEG dataset are stored in the.fif file format, with all stimuli information for each participant stored in the “derivatives/detailed_events” file. Thirdly, the task in NOD asked participants to actively indicate whether each presented image was animate or inanimate, which differs from the tasks used in the THINGS (detecting a catch image) and NSD (detecting a new image). Since the task context has potential to affect the neural representations of objects^55,56^, it is important to consider such differences when comparing object representations across datasets. Finally, since a participant was involved in experiments for all three modalities with the same stimuli, the later-collected EEG/MEG data may exhibit familiarity and repetition effects relative to the earlier-collected fMRI data^57,58^. Researchers should be aware of this when trying to integrate these datasets into an analysis.

## Data Availability

The code of the experimental design is available at https://github.com/BNUCNL/NaturalVisionProject; the code for preprocessing and technical validation is available at https://github.com/colehank/NOD-MEEG.
